# A Systematic Review of U.S.-Based Colorectal Cancer Screening Uptake Intervention Systematic Reviews: Available Evidence and Lessons Learned for Research and Practice

**DOI:** 10.3389/fpubh.2019.00145

**Published:** 2019-06-11

**Authors:** Belinda-Rose Young, Clement K. Gwede, Bria Thomas, Coralia Vázquez-Otero, Aldenise Ewing, Alicia L. Best, Claudia X. Aguado Loi, Dinorah Martinez-Tyson, Tali Schneider, Cathy D. Meade, Julie A. Baldwin, Carol Bryant

**Affiliations:** ^1^Department of Health Behavior, University of North Carolina at Chapel Hill, Chapel Hill, NC, United States; ^2^Prevention Research Center, College of Public Health, University of South Florida, Tampa, FL, United States; ^3^Moffitt Cancer Center & Research Institute and Morsani, College of Medicine, University of South Florida, Tampa, FL, United States; ^4^Health Sciences and Human Performance Department, University of Tampa, Tampa, FL, United States; ^5^Health Sciences Department, Northern Arizona University, Flagstaff, AZ, United States

**Keywords:** colorectal cancer screening (CRCS), evidence-based intervention (EBI), systematic review, effect size, research translation, evidence-based practice

## Abstract

**Background:** We examined colorectal cancer screening (CRCS) intervention effectiveness, through the effect sizes associated with: (1) screening modality, (2) intervention level (e.g., client-directed), and (3) intervention component (e.g. client reminders) within published CRCS intervention systematic reviews (SRs).

**Methods:** A search of peer-reviewed CRCS SRs that were written in English was employed utilizing five databases: CINAHL, Cochrane Library, rTIPS, PubMed, and PsycINFO EBSCOHOST. SRs that included CRCS interventions with a randomized controlled trial, quasi-experimental, or single arm design were eligible. Data on effect sizes by screening modality, intervention level, and intervention component were extracted and synthesized.

**Results:** There were 16 eligible CRCS intervention SRs that included 116 studies published between 1986 and 2013. Reviews organized data by CRCS screening modality, or intervention component. Effect size reporting varied by format (i.e., ranges, medians of multiple studies, or effect size per study), and groupings of modalities and components. Overall, the largest effect sizes were for studies that utilized a combination of colonoscopy, fecal occult blood test (FOBT), and sigmoidoscopy as screening options (16–45 percentage point difference).

**Conclusions:** Evidence suggests that CRCS interventions which include a combination of screening modalities may be most effective. This is the first SR to examine effect sizes of published CRCS SRs. However, because some SRs did not report effect sizes and there were tremendous variability reporting formats among those that did, a standard reporting format is warranted. Synthesizing findings can contribute to improved knowledge of evidence-based best-practices, direct translation of findings into policy and practice, and guide further research in CRCS.

## Introduction

### Rationale

Colorectal cancer (CRC) is the second leading cause of cancer-related deaths in the U.S. (1, 2). Despite steady decreases in CRC incidence and mortality, screening modalities are still markedly underutilized among some populations. Racial/ethnic minorities, medically underserved, and rural residents experience the highest CRC mortality rates in the U.S.(3). In addition, *The Guide to Community Preventive Services* (The Community Guide) identifies a number of evidence gaps for effective colorectal cancer screening (CRCS) interventions (4–10).

### Objectives and Research Question

On the hierarchy of evidence, systematic reviews represent the highest level of evidence and are often used by practitioners, policy-makers, and researchers to inform their work (11, 12). Several systematic reviews that examine CRCS interventions exist; however, the screening modality (i.e., type of screening test), priority population, and intervention level (i.e., client, provider, or system-directed) and component (i.e., behavior change strategy) vary widely. In this systematic review of systematic reviews, we sought to answer the question: what are the effective evidence-based interventions for CRCS, their effect size, and their characteristics? We synthesize evidence from published systematic reviews of CRCS interventions to provide a comprehensive and coherent picture of what is known, *and* to identify gaps in knowledge. The objective of this systematic review was to examine the effect sizes of CRCS interventions by (1) screening modality, (2) intervention level, and (3) intervention component.

In this systematic review, we examine published systematic reviews of interventions whose primary or secondary outcome was increased CRCS. To identify CRCS best-practices and address evidence gaps, we aimed to abstract the effect size(s) associated with the interventions. Through this review we, indirectly and to some extent, also assessed the quality of effect size reporting by CRCS systematic reviews more largely. The synthesis of evidence from this paper benefits the field by undergirding effective CRCS policy and practice efforts, ultimately leading to better patient care.

## Methods

### Search Strategy

**Figure 1:**
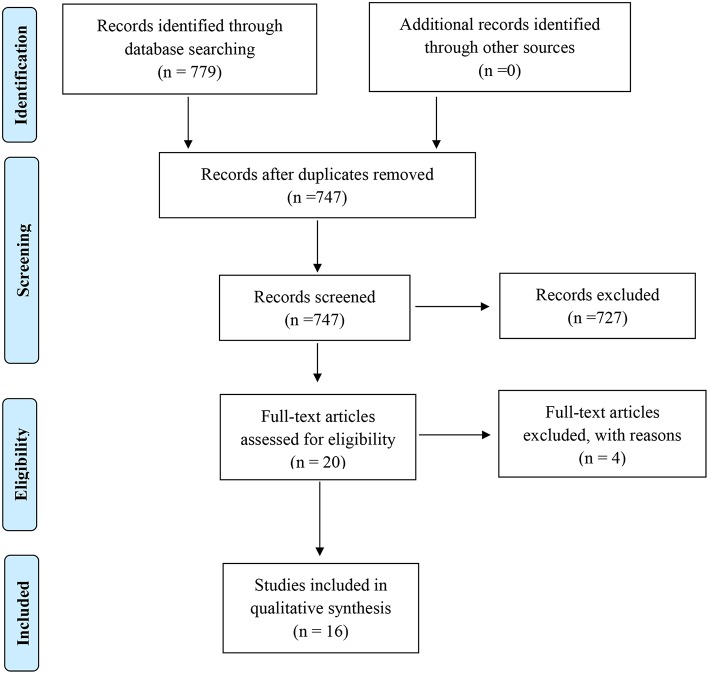
Flow diagram of the studies retrieved for the review.

A comprehensive search of published CRCS intervention systematic reviews was conducted, adhering to the Cochrane Collaboration guidelines (Figure 1) (13). In collaboration with experienced clinical research librarians, we performed a wide search in electronic databases (CINAHL, rTIPS, PubMed, Cochrane Library, and PsycINFO EBSCOHOST), and reviewed the reference section of each systematic review to see if they listed additional published CRCS systematic reviews. Among the electronic databases, PubMed included e-publications (i.e., “Ahead of print citations”), which decreased the risk of missing potential publications (14).

Our search aimed to locate any published systematic review that focused on improving CRCS through client, provider, and/or system-directed interventions. Search terms included (1) database-specific terms (e.g., MeSH terms) (Table 1) and identified key words for the databases that use booleans, or (2) only keywords for databases that do not have database-specific terms (e.g., rTIPS, Cochrane Library). The keywords were informed by the literature and added to by the healthcare librarians and experts in the field.

**Table 1:** Syntax for identifying CRCS intervention-focused systematic reviews within PubMed.

(((((systematic review^*^[tiab] OR “systematic review” OR meta-analysis OR review[tiab]))) AND ((((mass screening OR diagnosis OR early detection OR early detection of cancer OR forecasting OR catch OR check OR colonoscopy OR detect OR determine OR endoscopy OR examine OR FBOT OR fecal immunochemical test OR FIT OR filter OR find OR identity OR inspect OR investigate OR safeguard OR search OR sigmoidoscopy OR spot OR stool dna OR track OR uncover))) AND ((((tumor OR tumor OR polyps OR cancer OR neoplasms OR neoplasm OR carcinoma OR carcinomas OR neoplasia OR neoplasias OR neoplastic OR CRC OR colorectal neoplasms OR colonic neoplasms OR colonic cancer OR colorectal neoplasia OR rectal neoplasms OR anus neoplasms OR sigmoid neoplasms OR colorectal cancer OR rectal cancer OR sigmoid cancer OR colon cancer OR anal cancer OR sigmoid colon cancer OR recto-sigmoid cancer OR sigmoid rectal cancer))) AND ((colon OR colorectal OR rectum OR rectal OR anal OR anus OR sigmoid OR colon, sigmoid OR rectal sigmoid)))))) AND (screen^*^[tiab] AND (increas^*^[tiab] OR promot^*^[tiab])).

### Participants, Interventions, and Comparators

Eligible systematic reviews included those that were published in English, included studies conducted within the U.S. and/or its territories, utilized a RCT, quasi-experimental, or single arm intervention design, and whose primary or secondary outcome was CRCS uptake with any of the established screening modalities recommended by the U.S. Preventive Services Task Force (15). Studies were restricted to U.S.-based studies written in English in order to facilitate comparison among a more homogeneous group (e.g., federal laws, governing bodies, territories subject to recommendations by U.S. Preventive Services Task Force). Articles that solely focused on improving intentions to be screened were not included. Ineligible systematic reviews included: (1) all other review types (e.g., narrative literature reviews); or (2) systematic reviews that did not include any of the aforementioned design types or whose primary or secondary outcome was not CRCS uptake. Since our aim was to better understand the scope of existing published literature, we did not include gray literature.

The eligible CRCS modalities included: colonoscopy, sigmoidoscopy, CT colonography or virtual colonoscopy, double contrast barium enema (DCBE), or any stool test (i.e., DNA, fecal immunochemical test [FIT], fecal occult blood test [FOBT], high sensitivity guaiac FOBT [gFOBT]) as recommended by the U.S. Preventive Services Task Force (15). All other forms of CRCS were excluded from this review.

The research team co-developed a standardized review form to assess potential study eligibility; and based on the title and abstract, potentially eligible systematic reviews were selected by the primary reviewer using that form. Both the primary and secondary reviewer searched the full-text of each potentially eligible systematic review. If there were doubts concerning whether the systematic review met the eligibility criteria, the article was brought to the larger research team. The team then used the review form to systematically examine the article in question. Overall, at least two research team members assessed each systematic review for eligibility. The reference sections of the eligible systematic reviews were examined (i.e., hand-searched) to determine if any additional reviews were potentially eligible.

### Data Sources, Studies Sections, and Data Extraction

The research team was interested in gathering evidence of effective interventions more generally; therefore, we did not employ the entire PICO (participants, intervention, comparison, and outcome) method—which has been used within systematic reviews to identify components of clinical evidence. Employing the entire PICO method would have limited our understanding of available evidence since it would require us to hone in on a specific population (16–18). However, the portions of PICO that we did utilize were intervention and outcome—that is, systematic reviews had to include intervention studies whose primary or secondary outcomes centered on an increase in CRCS as an outcome of the intervention.

A data abstraction form was developed to assess and log the characteristics of the eligible systematic reviews and the effect sizes of the studies included within the systematic reviews. The items collected in the data abstraction form included: author(s); year of publication; eligibility criteria for publication; number of studies within the publication; study setting; and effect sizes (e.g., median, ranges) by screening modality, intervention level(s), and intervention component(s). This form was created, reviewed and pretested by three authors prior to abstraction. After extensive training and discussion, one author abstracted the data, while another independently reviewed all data. Minor disagreements were resolved by an arbitrator. The individual articles were not assessed for data quality as they were already reported within the respective systematic review.

### Data Analysis

The effect size is a measure to describe the magnitude of effect, which is a quantification of the difference between two groups in the observed outcome (here, screening uptake). The effect size can also be described as the percentage point difference between two groups, or the percent change from baseline of a single group. Effect sizes are often more telling than tests of statistical significance (*p*-values) (19). This is because they show the magnitude of difference between two groups, as opposed to stating that there was an observed difference (19). Thus, the effect size can help us to prioritize one effective intervention over another.

Data were synthesized in two ways. First, data were synthesized more generally by screening modality, intervention level, and intervention component. Next, to address CRCS evidence gaps as identified by The Community Guide, data were synthesized around the seven gaps (as detailed in the results section).

## Results

### Study Selection and Characteristics

Out of 747 systematic reviews screened from five databases, 16 systematic reviews met the inclusion criteria (2.14%) (Figure 1). Most reviews were ineligible because they did not include CRCS studies, or because they did not include a behavioral intervention (e.g., focused on attitudes and beliefs of certain cancer risk factors). The 16 systematic reviews contained 206 studies, of which 116 were unique. The publication dates of the studies included within the 16 systematic reviews ranged from 1986 to 2013, representing over 27 years' worth of knowledge. Of the 16 eligible systematic reviews, 12 reported information about effect sizes.

Each of the 16 systematic reviews varied in their inclusion criteria used to determine study eligibility. Though some eligibility criteria could be inferred based upon the descriptions of the studies examined, we did not denote the eligibility criteria if they were not explicitly stated within the systematic review. The most frequent criteria (Table 2) included *research design restriction* (*n* = 9; 56%), an increase in *CRCS as the primary outcome* (*n* = 7; 44%), and *CRCS behavioral intervention* (*n* = 16). Six systematic reviews, published between 2003 and 2012, containing studies between 1986 and 2007, required eligible studies to be the *primary scientific publication*, and not a secondary article of the same study. Six systematic reviews published between 2008 and 2012 had a study publication date range restriction (collectively, 1997–2010) for their inclusion criteria.

**Table 2:** Frequent inclusion criteria across the 16 systematic reviews.

**References**	**Publication year range^**a**^**	**Publication year/range restriction^**b**^**	**Primary scientific publication^**c**^**	**CRCS behavioral intervention^**d**^**	**Research design restriction^**e**^**	**Quality of execution^**f**^**	**Increase in CRCS as primary outcome^**g**^**	**Race/ethnicity^**h**^**
Powe and Finnie (20)	1995		X	X				
Baron et al. (34)	1992–2001		X	X	X	X	X	
Baron et al. (35)	1986–2004		X	X	X	X	X	
Sabatino et al. (21)	1986–1998		X	X	X	X	X	
Ward et al. (22)	2000-2007	X 1/2000-8/2007		X				X
Baron et al. (23)	1989–2002		X	X	X	X	X	
Holden et al. (24)	2000–2009	X 1/1998-9/2009		X				
Morrow et al. (25)	2001–2009			X	X		X	X
Powe et al. (26)	2000–2007	X 1/2000-12/2008		X				X
Brouwers et al. (27)	2005–2010	X 1/2004-12/2010		X	X		X	
Ferroni et al. (28)	1986–2009	X 1/1999-12/2009		X				
Gonzalez et al. (29)	2005–2011			X	X			X
Rawl et al. (30)	1997–2008	X 1/1997-12/2007		X	X			
Sabatino et al. (31)	1986–2007		X	X	X	X	X	
Oh and Jacobsen (32)	2009			X				X
Muliira and D'souza (33)	2005–2013			X				
TOTAL	1986–2013	6	6	16	9	5	7	5

a *Publication year range = the year range of studies included within each SR*;

b *Publication year/range restriction = SR authors restricted their article search to a specific time period*;

c *Primary scientific publication = first publication from a study*;

d *CRCS behavioral intervention = an intervention aimed at increasing CRCS*;

e *Research design restriction = denotes SR authors restricted eligible studies to those that included research designs of interest to the authors*;

f *Quality of execution = SR authors determined whether a study met a certain level of quality*;

g *Increase in CRCS as primary outcome = indicates that an increase in CRCS could not be a byproduct or secondary focus of the study—it had to be the main outcome*;

h *Specific race/ethnicity = SR only included studies that restricted participants' races and/or ethnicities*.

Less than half included restrictions on *quality of study execution* (*n* = 5; 31%), *race/ethnicity* (*n* = 5; 31%), *intervention level or component* (*n* = 2, 13%), and *age range* (*n* = 2, 13%). A minority of systematic reviews (*n* = 5) included a restriction that was not used within other reviews: specific screening modality; cultural appropriateness of intervention; intervention sample size; setting of intervention; and study effect sizes reported (25, 26, 28, 29, 33).

### Synthesized Findings

Across the 16 eligible systematic reviews, findings included interventions at the three levels described by *The Community Guide*. Briefly, these are client-directed/oriented (e.g., small-media, mass-media, education/counseling, structural barriers), provider-directed/oriented (e.g., assessment and feedback, computer generated reminders), and system-directed/oriented (e.g., shared decision making, systematic screening, patient navigator, referral system). Many systematic reviews focused on one specific intervention level, mostly client-directed (*n* = 9; 56.25%) (20, 25, 27–29, 32–35). However, five additional systematic reviews (31.25%) included client-directed and at least one other intervention level (22, 24, 26, 30, 31). Table 3 describes the intervention level(s) and component(s) of each eligible SR. The interventions took place in several settings (e.g., health clinics, churches, community events, homes).

**Table 3:** Characteristics of eligible systematic reviews.

**References**	**Number of studies included that met our eligibility**	**Study design(s)**	**Intervention level(s)**	**Intervention component(s)**
Powe and Finnie (20)	1	Single arm intervention	Client-directed	Small-media
Baron et al. (34)	7	RCT	Client-directed	Structural barriers
Baron et al. (35)	14	1. RCT 2. Quasi-experimental 3. Single arm intervention	Client-directed	1. Client reminders 2. Small-media 3. Mass media 4. Group education 5. One-on-one education
Sabatino et al. (21)	4	1. RCT 2. Single arm intervention	Provider-directed	1. Provider assessment and feedback 2. Provider incentives
Ward et al. (22)	8	1. RCT 2. Single arm intervention	1. Client-directed 2. Provider-directed	1. Small-media 2. Group education
Baron et al. (23)	6	RCT	Provider-directed	Provider reminder
Holden et al. (24)	23	1. RCT 2. Quasi-experimental 3. Single arm intervention	1. Client-directed 2. Provider-directed 3. System-directed	1. Structural barriers 2. Client reminders 3. Small-media 4. Provider reminder 5. Patient navigator 6. Group education
Morrow et al. (25)	15	RCT	Client-directed	1. Small-media 2. One-on-one education 3. Group education
Powe et al. (26)	12	1. RCT 2. Single arm intervention 3. Quasi-experimental	1. Client-directed 2. Provider-directed	1. Small-media 2. Client reminders 3. Provider reminders 4. Group education
Brouwers et al. (27)	39	RCT	Client-directed	1. Structural barriers 2. Client reminders 3. Client incentives 4. Small-media 5. Provider feedback and assessment 6. Group education 7. One-on-one education
Ferroni et al. (28)	3	RCT	Client-directed	1. Small media 2. One-on-one education
Gonzalez et al. (29)	5	1. RCT 2. Single arm intervention	Client-directed	1. Client reminders 2. Small media 3. One-on-one education
Rawl et al. (30)	34	RCT	1. Client-directed 2. Provider-directed 3. System-directed	1. Structural barriers 2. Client reminders 3. Patient navigator 4. Small-media 5. Group education 6. One-on-one education
Sabatino et al. (31)	25	1. RCT 2. Quasi-experimental 3. Single arm intervention	1. Client-directed 2. Provider-directed	1. Structural barriers 2. Client reminders 3. Patient navigator 4. Provider incentives 5. Provider assessment and feedback 6. Group education 7. One-on-one education
Oh and Jacobsen (32)	1	Quasi-experimental	Client-directed	1. Patient navigator 2. Client reminders 3. Structural barriers
Muliira and D'souza (33)	15	1. RCT 2. Quasi-experimental 3. Single arm intervention	Client-directed	Patient navigator

### Effect Size

Authors of the published CRCS systematic reviews organized the papers by intervention component or screening modality, and then described the studies accordingly. Some were individually described, while others were presented in an aggregated format. In most cases, the systematic review authors described the effect sizes in the same way they described study characteristics—individually or aggregated. If stated, the effect sizes were described as: (1) the median difference in percentage points (compared to the control group); (2) range of percentage point difference; or (3) both.

Because some systematic reviews included studies at different intervention levels and varied in intervention components (i.e., behavior change strategies), it was possible for a single systematic review to be utilized multiple times in our analysis. For instance, a systematic review could contain studies that included a client-directed group education intervention, and other studies that were provider-directed, with the intervention component being provider incentives. Thus, one systematic review could provide effect size data for multiple screening modalities, intervention levels, and/or intervention components. We thought this was the strongest approach to analyzing the data, because it allowed us to accurately reflect the breadth of information and prevalence of effect size reporting. Ultimately, we examined effect sizes by: (1) screening modality, (2) intervention level, and (3) intervention component by intervention level.

### Effect Size by Screening Modality

Among systematic reviews that assessed the effect size by screening modality, data from studies were reported in the context of a particular screening modality only, a combination of modalities, or both (i.e., included both a predetermined group of modalities compared and a singular option) (Table 4). Eight systematic reviews included a combination(s) of screening modalities in their analysis. The frequency of systematic reviews that contained a modality within a particular combination is as follows: colonoscopies (*n* = 6), sigmoidoscopy (*n* = 6), FOBT (*n* = 6), FIT (*n* = 3), DCBE (*n* = 3), and endoscopic (*n* = 2). Seven systematic reviews included at least one study that did not report the screening modality, but rather reported only the difference in screening uptake. The effect size by screening modality varied both within modality and across modalities.

**Table 4:** Magnitude of effects medians and range by screening modality (as a screening outcome).

**Screening modality**	**Number of SRs including studies with the Screening Modality of interest^**a**^**	**Median^**b**^ (reported within a single SR)**	**Range^**c**^ (reported within a single SR)**	**Comments**
Combination (FOBT & Sigmoidoscopy)	2	13, 15.3	12–23 PP^d^	
Combination (Colonoscopy & Sigmoidoscopy)	1	NR^e^	NR^e^	
Combination (Colonoscopy, FOBT & Sigmoidoscopy)	4	8.9, 36.9	16–45 PP^d^	Range: 3 SRs did not mention Median: 2 SRs did not mention
Combination (Colonoscopy, FIT & FOBT)	1	NR^e^	NR^e^	
Combination (Colonoscopy, DCBE, FIT, FOBT, & Sigmoidoscopy)	1	NR^e^	NR^e^	
Combination (Colonoscopy, DCBE, & Sigmoidoscopy)	1	0.5	0-6 PP^d^	
Combination (DCBE, Endoscopic & FOBT)	1	NR^e^	(-0.1) to 2.8 PP^d^	
Combination (Any CRCS test)	2	NR^e^	1-11 PP^d^	Range 1 SR did not mention Median: 2 SRs did not mention
FOBT alone	8	11.5, 12.7, 4.4, 16.1	(−13) to 37 PP^d^	Range: 6 SRs did not mention

a *Multiple studies within a SR (i.e., contains the number of SRs that contained a study with the restricted screening modality as an option, thus total number does not equate to 16 nor 116)*;

b *Median, average difference in percentage points (compared to the control group) across different studies*;

c *Range, range of percentage point difference across different studies*;

d *PP, percentage points*;

e *NR, Not reported*.

Half of the systematic reviews (*n* = 8) included a section of studies that used FOBT as the sole screening outcome and, among available data, reported a percentage point difference ranging from −13 to 37%. It is important to note that of those eight systematic reviews, only two reported a range; and half reported a median percentage point increase (4.4, 11.5, 12.7, and 16.1). The systematic reviews that did not include an effect size typically indicated that there was an “increase in screening.”

### Effect Size by Intervention Level

We also assessed the effect size by intervention level. Fourteen of the 16 systematic reviews included at least one study that intervened at the client-level (−13 to 42 percentage points). Seven systematic reviews included at least one study that intervened at the provider-level (−0.1 to 23 percentage points) (21–24, 26, 30, 31). Two systematic reviews included a study that intervened at the system-level (7–28 percentage points) (24, 30).

### Effect Size by Intervention Component

Among the client-directed interventions, the most frequent intervention component was one-on-one education (*n* = 4) (effect sizes not reported), client-reminders (*n* = 3) (0.0–0.6 percentage points), and small-media (*n* = 3) (effect sizes not reported). However, nine SRs presented a combination of client-directed intervention components—most of which included client-reminders (*n* = 5) (−7 to 42 percentage points). The least frequent component, overall, was the use of patient navigators (*n* = 1) (effect sizes not reported).

Fewer systematic reviews included either provider-directed (*n* = 7), or system-directed interventions (*n* = 2). Within provider-directed interventions, provider reminder (*n* = 2) (15.3 percentage point median reported in one of the systematic reviews), provider assessment and feedback (*n* = 2) (13 to 45 percentage point median range), and provider incentives (*n* = 2) (−0.1 to 2.8 percentage points reported in one of the systematic reviews) were the most common. The system-directed intervention components included patient navigators and a patient-referral system (7–28 percentage points) and/or a multi-component office-based intervention (e.g., checklists, chart stickers, audits) (effect size not reported).

### Evidence for the Community Guide

The Community Guide has identified seven CRCS intervention areas that currently have “insufficient evidence.” These intervention areas span multiple intervention levels and include various intervention components and screening modalities. We use data from our systematic review to corroborate and/or inform the seven CRCS intervention areas gaps (Table 5).

**Table 5:** The Community Guide's Areas of Insufficient CRCS evidence identified by this study.

**CRCS Community Guide Insufficient Evidence Area**	**Evidence finding year**	**New evidence**	**Nature of new evidence finding**
Provider assessment and feedback on increasing non-FOBT screening	2008	None	N/A
Client reminders on increasing non-FOBT screening	2008	(27)	Number of studies:4 Range: 0PP –6PP^a^ Screening modality: 1. FIT (1 study) 2. Flexible Sigmoidoscopy (1 study) 3. Colonoscopy (2studies)
		(31)	Number of studies:2 Range: 0PP –6PP^a^ Screening modality: 1. Barium Enema 2. Flexible Sigmoidoscopy 3. Colonoscopy
Small-media on increasing sigmoidoscopy, colonoscopy, or DCBE	2005	(27)	Number of studies:1 Effect size:11.2PP^a^ Screening modality:Colonoscopy
Client incentives to increase CRCS (for any screening modality)	2010	None	N/A
Reducing out of pocket costs to increase CRCS	2009	(27)	Number of studies:1 Effect size: 4.2PP^a^ (not significant) Screening modality: Notreported
Group education to increase CRCS	2009	(27)	Inconsistent findings^b^ Number of studies:2 Range: Not reported Effect size: Notreported
		(24)	Inconsistent findings^b^ Number of studies:2 Range: Not reported Effect size: Notreported
		(31)	Inconsistent findings^b^ Number of studies:2 Range:−13PP −37PP^a^ Effect size: 4.4PP^a^
Mass media to increase CRCS	2009	(31)	Negative direction Number of studies:1 Range: Not Reported Screening modality: 1. FOBT (-4.7 PP^a^) 2. Proctoscopy (-8.0 PP^a^)

a *PP, percentage point difference*.

b *For the purposes of our review, inconsistent findings refers to one or more studies with results in a direction opposite the primary listed study within the systematic review's subsection*.

### Gap 1: Impact of Provider Assessment and Feedback on Increasing Non-FOBT Screening

We found two systematic reviews that included information about provider assessment interventions (21, 31). Both reviews included the same three studies. However, only one of the studies included a screening modality other than FOBT-Sigmoidoscopy-for which there was no CRCS uptake change (36).

### Gap 2: Impact of Client Reminders on Increasing Non-FOBT Screening

Five of the systematic reviews included studies with a client reminder intervention component; however, two of those included FOBT as the screening modality outcome (29, 35). Of the remaining three systematic reviews, two included studies (*n* = 11 studies; *n* = 33 studies) with multiple screening modality outcomes (27, 30). One systematic review found significant, positive percentage point change (3–40.8) among four studies where the screening modality outcome was FIT, flexible sigmoidoscopy, or colonoscopy; with colonoscopy studies being associated with the highest percentage point differences (11.7 to 40.8) (27). Of the 33 studies included in the second systematic review, 10 included an outcome of FOBT and 16 included an outcome of “any CRCS test” (30). The remaining seven used endoscopy procedures (flexible sigmoidoscopy, or colonoscopy), five of which reported to have significant intervention effects; however, due to variable reporting styles (“actual percent completing the test, to percent increase from baseline, to odds ratios,” p.177), authors indicated that comparisons were unable to be made between the studies. The last applicable systematic review included two studies with barium enema, flexible sigmoidoscopy, or colonoscopy as the outcome (31). Collectively, these studies contained five intervention arms, and showed a median increase of 0.5 percentage points (range: 0.0 to 6.0).

### Gap 3: Impact of Small Media on Increasing Sigmoidoscopy, Colonoscopy, or DCBE

Seven systematic reviews included small media interventions; however, three did not state the testing outcome of the studies, and one included FOBT as the screening modality outcome. Three systematic reviews remained that included the test outcomes of interest (25–27). One systematic review included nine studies that had the outcome option of “any screening modality,” whereas the last study only included colonoscopies as an outcome (11.2 percentage points) (27). Another systematic review included seven studies that used small media, but the effect estimate chosen (odds ratio) was only available for two of the studies, for which multiple screening modalities were an option (25). While the last systematic review included studies with the desired intervention and screening modality, they either included multiple intervention components, thus muddying the understanding of small-media's direct impact, or no effect estimates were provided (26).

### Gap 4: Client Incentives to Increase CRCS for Any Screening Modality

No studies in the 16 systematic reviews that we examined discussed this intervention component.

### Gap 5: Reducing Out-of-Pocket Costs to Increase CRCS

One systematic review included this intervention component; however, the findings were not significant (4.2 percentage points) (27).

### Gap 6: Impact of Client Education for Increasing CRCS

Six systematic reviews included studies with group education as an intervention component; however, one indicated that there was “insufficient evidence” (35). Two systematic reviews included studies that employed multiple components within their intervention, thus making it difficult to determine which component was most impactful (22, 25). Three systematic reviews included studies that only utilized group education as the intervention component, yet had inconsistent findings (24, 27, 31). For example, one systematic review included studies where the control group had a higher increase in CRCS than the intervention group; in another study (within the same SR) the opposite was true (27). Another systematic review included two group education intervention studies in their review, with one showing a negative effect (-7%) and the other a positive increase in screening (12%) (24). The last systematic review included two studies with a median of 4.4 percentage points, and a range of −13 to 37% (31). Our findings mirror those of *The Community Guide*—there is inconsistent evidence.

### Gap 7: Impact of Mass Media on CRCS

Only one systematic review included a study that utilized mass media as the intervention (31). Two screening modality options were available—FOBT and proctoscopy—and both yielded results in a negative direction (−4.7 and −8.0 percentage points, respectively). More evidence is needed about utility and feasibility of mass media CRCS interventions.

## Discussion

### Summary of Main Findings

Through this systematic review of systematic reviews, we were able to examine 116 CRCS studies and found that consistently, based on effect sizes, the most effective intervention characteristics were those that: provided clients with the option to select from a colonoscopy, FOBT, or sigmoidoscopy screening modality (16–45 PP); targeted systems through patient navigators or a patient-referral structures (7–28 PP); or intervened at the provider-level through provider assessment and feedback (13–45 PP). By seeking to understand the most effective CRCS evidence-based interventions and their characteristics, we were able to provide a synthesis of effect estimates by screening modality, intervention level, and intervention component. Furthermore, our findings help to fill in some of the gaps identified by *The Community Guide*, and reinforce what current evidence is needed.

The interpretation of individual or a group of studies within the context of the *totality* of evidence can provide better decision aids, inform guidelines, and advance health policies. The results of individual studies may be misleading due to potential variation in findings and interpretation (37). Thus, to help inform clinical and research decisions, we synthesized all available CRCS intervention data from eligible systematic reviews. We assessed interventions to increase CRCS, which contributes to improved knowledge of the impact of screening modality, intervention level, and/or intervention component on CRCS uptake. Using effect sizes (rather than *p*-values) improves interpretation of intervention results because it facilitates an understanding of the magnitude and direction of the significance. Ultimately, this enables consistent evidence-based decision-making.

### Strengths

This systematic review included a comprehensive search strategy, guided by expert opinion, and utilized five separate electronic databases. We followed Cochrane Collaboration guidelines and used a two-reviewer approach with arbitration as necessary, and larger research team input. We also included 27 years' worth of published data through 16 systematic reviews that were nuanced, yet consistent in purpose.

For the systematic reviews that reported summary statistics (*n* = 12), we were able to either corroborate conclusions or add evidence to six of the seven gaps identified by *The Community Guide*. These findings represent an important step toward advancing the CRCS intervention evidence base knowledge. Although four of the 16 systematic reviews did not include summary statistics, our analysis of these provided valuable evidence on the types of CRCS interventions being implemented, including screening modality, intervention level, and intervention components.

## Limitations

Although our systematic review included 116 unique studies, our purpose was to get a better understanding of the effect sizes and the best evidence by abstracting data at the systematic review level. We also, in a sense, were evaluating the structure of, and what was missing from, CRCS intervention systematic reviews. Findings reported were based on available evidence. Because effect sizes were not reported within every category (e.g., intervention component), it is possible that the ranges for a particular category could be wider.

While we were able to quantify that the 16 systematic reviews included 116 unique studies by reviewing the reference sections, we are not always told (within the systematic review itself) which studies were examined to answer each of their research questions. Because we abstracted data at the systematic review level, it is possible that some systematic reviews included the same studies (to answer similar research questions), thus resulting in more evidence for a particular category (e.g., screening modality, intervention level, intervention component) or any of its sub-categories.

Finally, since the focus of this systematic review was to examine the evidence at the systematic review level, the reviewers did not examine the studies within each systematic review. Thus, we cannot determine if the lack of effect size reporting from four systematic reviews was due to the systematic review authors' omission, or the lack of reporting by the study authors. This information would be helpful for prioritizing future reporting directives.

### Future Directives and Implications for Research, Policy, and Practice

This review of systematic reviews has broadened our understanding of prevailing evidence. Gaining a deeper insight into what CRCS interventions are truly promising provides a sound basis for clinical research translation into best practices and further research. In addition, it decreases the likelihood of wasting resources, and, most importantly, can lead to an increase in CRCS among individuals with greatest risk. We provided data that begins to fill some of the gaps in the CRCS evidence base identified by *The Community Guide*. For example, we found that while most interventions were aimed at the client-directed level, the interventions conducted at system and provider-levels had the most marked effects. Further, interventions that offered another screening modality in addition to FOBT were often more effective than when FOBT was the sole option. Nonetheless, a number of questions remain about the evidence of effectiveness for certain intervention components and CRCS modalities. Additionally, questions about the reporting structure of CRCS systematic reviews emerged. For example, many systematic reviews reported a summary statistic (e.g., effect size median), making it easier for the reader to understand and apply the information. However, some systematic reviews did not, which made it more challenging to compare data within and across systematic reviews.

Future research should examine and propose criteria for reporting effect sizes for screening uptake in systematic reviews. If necessary intervention features are missing, then the use of the systematic review for program planning, research and funding decisions is less appreciable. Failure to publish effect sizes of studies included within a systematic review may lead to a lack of research uptake. Variance in effect size reporting among CRCS systematic reviews calls for the creation of minimum standards that could also be applied to systematic reviews more widely, possibly leading to greater uptake (37). Such standards may result in a trickle-down effect of increased effect size reporting within publications of individual studies. These enhancements will aid in research interpretation, better practice decision-making, and overall improved care.

## Conclusion

This study provides a systematic review of available peer-reviewed evidence on CRCS interventions. Findings suggest that CRCS interventions that utilized a combination of colonoscopy, FOBT, and sigmoidoscopy screening modalities, or targeted systems through patient navigators or a patient-referral structures, or intervened at the provider-level through provider assessment and feedback were most effective. Evidence from this study provides public health stakeholders with the information needed to make informed decisions about potential interventions to increase CRCS interventions. Findings also illuminate gaps in knowledge that should be prioritized by future research.

## Author Contributions

BY and CV-O contributed to the design of the study, data acquisition, data interpretation, manuscript development and revisions, and approved the final version of the submitted manuscript. CG, BT, AE, CA, DM-T, CM, JB, and CB contributed to the design of the study, data interpretation, manuscript development and revisions, and approved the final version of the submitted manuscript. AB contributed to data interpretation, manuscript revisions, and approved the final version of the submitted manuscript. TS contributed to the design of the study, manuscript development and revisions, and approved the final version of the submitted manuscript.

### Conflict of Interest Statement

The authors declare that the research was conducted in the absence of any commercial or financial relationships that could be construed as a potential conflict of interest.
